# Supplementary material for: Discovery of antitumor lectins from rainforest tree root transcriptomes
Source: PLoS One. 2020 Feb 25;15(2):e0229467. doi: 10.1371/journal.pone.0229467 (PMC7041804; doi:10.1371/journal.pone.0229467)
Supplement: S1 Fig — (DOCX) [file pone.0229467.s001.docx]

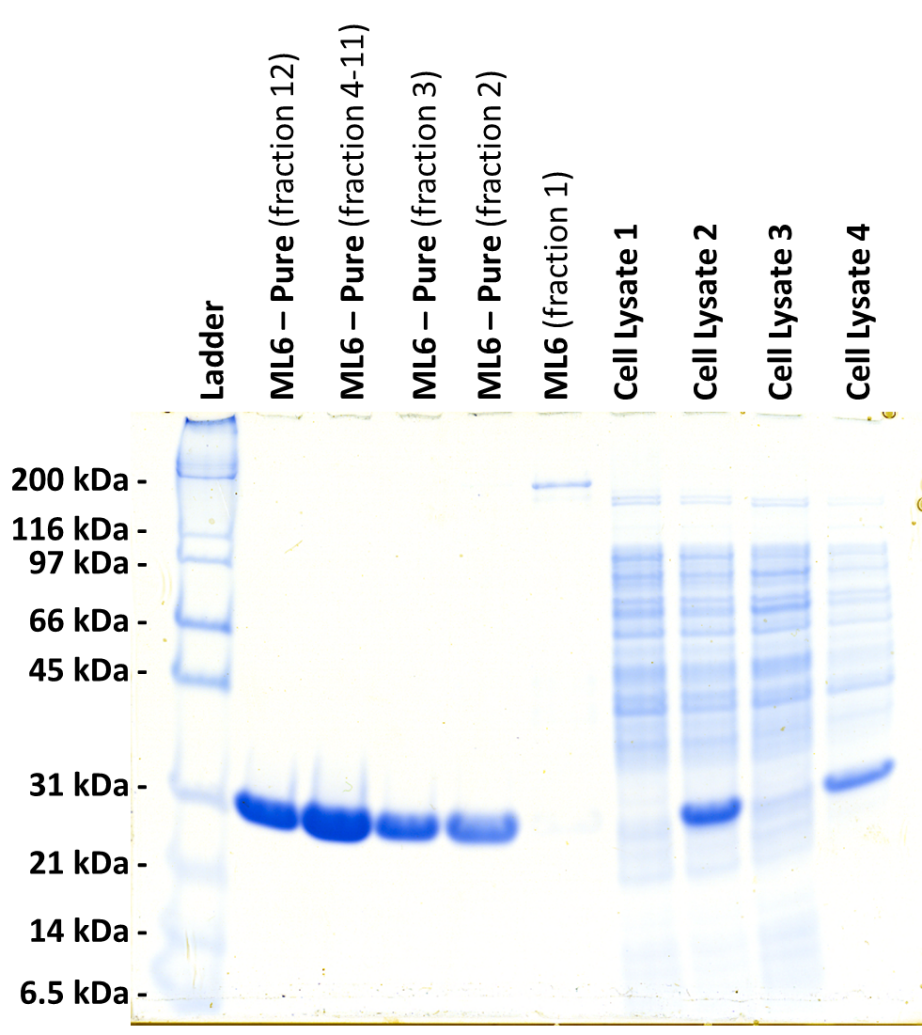


S1 Fig. SDS-PAGE gel of purified recombinant ML6 lectin. Representative separation shown. Purified fractions 3 – 12 are pooled to obtain the pure protein for studies. Whole cell lysates are shown for reference.
