# Supplementary material for: Discovery of antitumor lectins from rainforest tree root transcriptomes
Source: PLoS One. 2020 Feb 25;15(2):e0229467. doi: 10.1371/journal.pone.0229467 (PMC7041804; doi:10.1371/journal.pone.0229467)
Supplement: S2 Fig — Representative isothermal titration calorimetry data of ML6 (163 μM) in the presence of a 10-fold excess of mannose haptens (1.63 mM). Kd = 1.5 μM. (DOCX) [file pone.0229467.s002.docx]

S2 Fig. ITC binding. Representative isothermal titration calorimetry data of ML6 (163 µM) in the presence of a 10-fold excess of mannose haptens (1.63 mM). K_d_ = 1.5 µM.
