# Supplementary material for: Discovery of antitumor lectins from rainforest tree root transcriptomes
Source: PLoS One. 2020 Feb 25;15(2):e0229467. doi: 10.1371/journal.pone.0229467 (PMC7041804; doi:10.1371/journal.pone.0229467)
Supplement: S3 Fig — Hyphae of a plant pathogenic fungus, Mycosphaerella zeae, grow profusely in (A) untreated media, but are (B) stunted following 24 hour treatment with ML6 (500nM). Hyphae remained stunted for 19 days in the presence of ML6, after which the experiment was ended. (DOCX) [file pone.0229467.s003.docx]

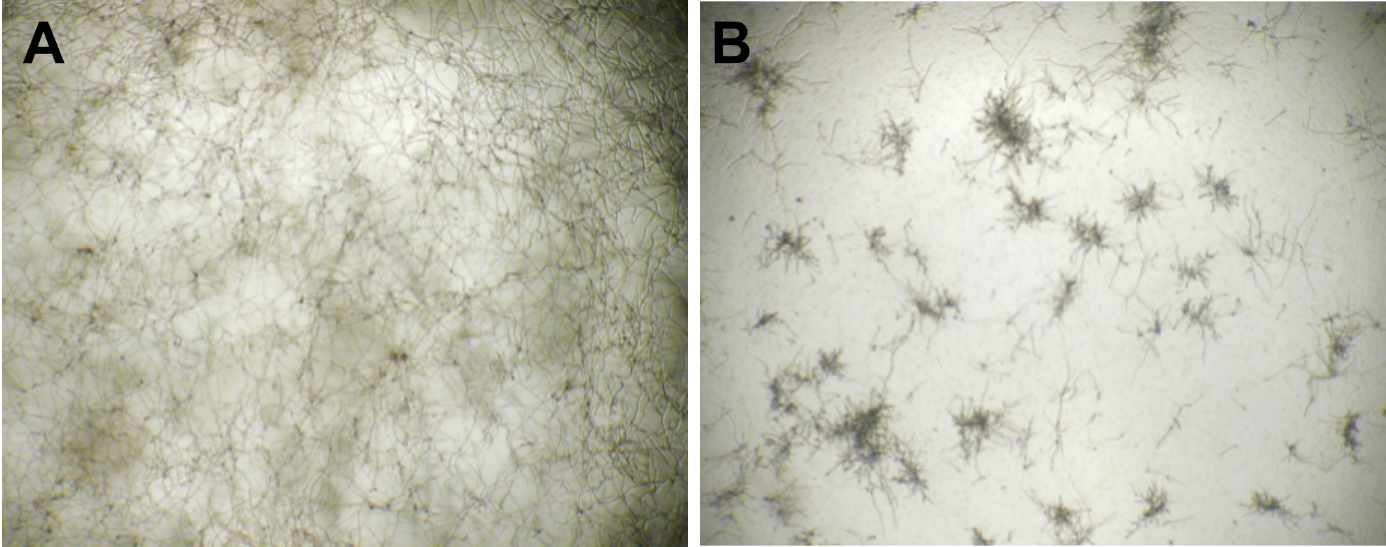


S3 Fig. Anti-mycologic activity of ML6. Hyphae of a plant pathogenic fungus, *Mycosphaerella zeae*, grow profusely in (A) untreated media, but are (B) stunted following 24 hour treatment with ML6 (500nM). Hyphae remained stunted for 19 days in the presence of ML6, after which the experiment was ended.
