# Supplementary figures and images for: Discovery of antitumor lectins from rainforest tree root transcriptomes
Source: PLoS One. 2020 Feb 25;15(2):e0229467. doi: 10.1371/journal.pone.0229467 (PMC7041804; doi:10.1371/journal.pone.0229467)

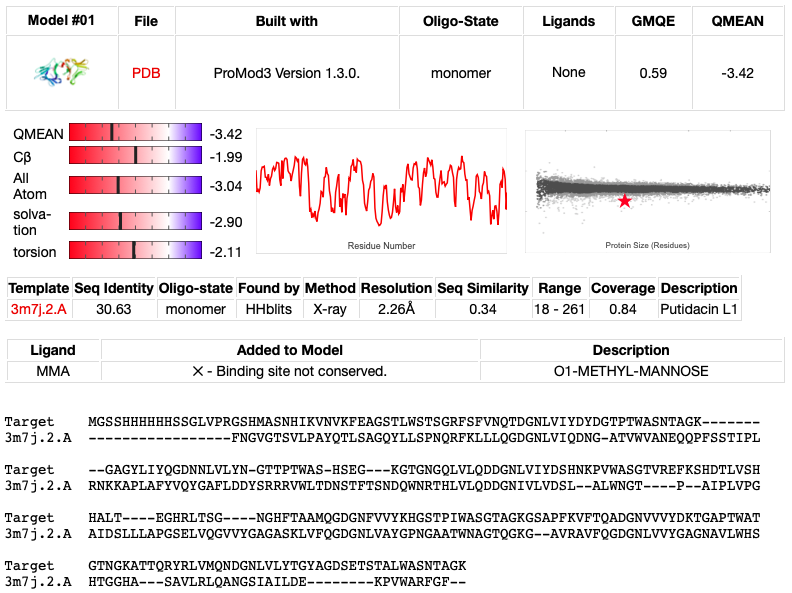


**S4 Fig. Report of target-template model chosen for ML6 homology model build.**

Supplement: S4 Fig — (DOCX) [file pone.0229467.s004.docx]
