# Supplementary material for: Discovery of antitumor lectins from rainforest tree root transcriptomes
Source: PLoS One. 2020 Feb 25;15(2):e0229467. doi: 10.1371/journal.pone.0229467 (PMC7041804; doi:10.1371/journal.pone.0229467)
Supplement: S5 Fig — Viability of A549 cells in the absence (dark red) or presence of free mannose haptens at a low (0.1 mM, red) and high (1 mM, pink) concentration of the sugar in culture media. (DOCX) [file pone.0229467.s005.docx]

S5 Fig. Competitive inhibition of ML6 activity. Viability of A549 cells in the absence (dark red) or presence of free mannose haptens at a low (0.1 mM, red) and high (1 mM, pink) concentration of the sugar in culture media.
