# Supplementary material for: Discovery of antitumor lectins from rainforest tree root transcriptomes
Source: PLoS One. 2020 Feb 25;15(2):e0229467. doi: 10.1371/journal.pone.0229467 (PMC7041804; doi:10.1371/journal.pone.0229467)
Supplement: S6 Fig — IC50 for three batches of recombinantly-expressed ML6 protein against the A549 lung carcinoma cell line. †IC50 could not be reached over the tested concentration range. (DOCX) [file pone.0229467.s006.docx]

****S6 Fig. Batch-to-batch variance of ML6 potency. IC_50_ for three batches of recombinantly-expressed ML6 protein against the A549 lung carcinoma cell line. ^†^IC_50_ could not be reached over the tested concentration range.
