# Supplementary material for: Discovery of antitumor lectins from rainforest tree root transcriptomes
Source: PLoS One. 2020 Feb 25;15(2):e0229467. doi: 10.1371/journal.pone.0229467 (PMC7041804; doi:10.1371/journal.pone.0229467)
Supplement: S7 Fig — CD spectra for batch 1 (●), 2 (■), and 3 (▲) of the recombinantly-expressed ML6 protein. (DOCX) [file pone.0229467.s007.docx]

S7 Fig. Circular dichroism analysis of ML6. CD spectra for batch 1 (●), 2 (■), and 3 (▲) of the recombinantly-expressed ML6 protein.
