# Supplementary material for: Discovery of antitumor lectins from rainforest tree root transcriptomes
Source: PLoS One. 2020 Feb 25;15(2):e0229467. doi: 10.1371/journal.pone.0229467 (PMC7041804; doi:10.1371/journal.pone.0229467)
Supplement: S8 Fig — Average cluster area of cell aggregates measured from microscopy images of A549 cells before (black bar) and after (open bar) a 1 hour incubation with 10 μM ML6. (DOCX) [file pone.0229467.s008.docx]

S8 Fig. ML6-mediated cell agglutination. Average cluster area of cell aggregates measured from microscopy images of A549 cells before (black bar) and after (open bar) a 1 hour incubation with 10 μM ML6.
