# Supplementary material for: Discovery of antitumor lectins from rainforest tree root transcriptomes
Source: PLoS One. 2020 Feb 25;15(2):e0229467. doi: 10.1371/journal.pone.0229467 (PMC7041804; doi:10.1371/journal.pone.0229467)
Supplement: S9 Fig — Fluorescent micrographs of A549 cell controls stained with antibody (1:1000 FITC-tagged Anti-6XHIS) in the absence of ML6 pre-treatment. (DOCX) [file pone.0229467.s009.docx]

S9 Fig. Antibody staining control images. Fluorescent micrographs of A549 cell controls
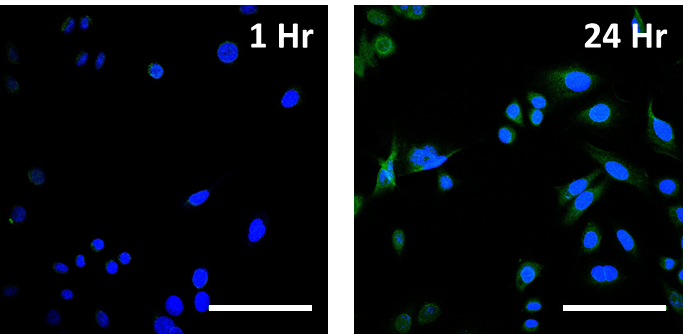
stained with antibody (1:1000 FITC-tagged Anti-6XHIS) in the absence of ML6 pre-treatment.
