# Supplementary material for: Discovery of antitumor lectins from rainforest tree root transcriptomes
Source: PLoS One. 2020 Feb 25;15(2):e0229467. doi: 10.1371/journal.pone.0229467 (PMC7041804; doi:10.1371/journal.pone.0229467)
Supplement: S1 Table — (DOCX) [file pone.0229467.s010.docx]

**S1 Table. Templates selected for ML6 model building**

| Template | Seq Identity | Oligo-state | QSQE | Found by | Method | Resolution  (Å) | Seq Similarity | Coverage | Description |
| --- | --- | --- | --- | --- | --- | --- | --- | --- | --- |
| 3m7j.2.A | 30.63 | monomer |  | HHblits | X-ray | 2.26 | 0.34 | 0.84 | Putidacin L1 |
| 4lea.2.A | 30.84 | monomer |  | HHblits | X-ray | 2.55 | 0.35 | 0.81 | Pyocin L1 |
| 4le7.2.A | 30.84 | monomer |  | HHblits | X-ray | 2.09 | 0.35 | 0.81 | Pyocin L1 |
| 4le7.1.A | 30.84 | monomer |  | HHblits | X-ray | 2.09 | 0.35 | 0.81 | Pyocin L1 |
| 1dlp.1.A | 33.18 | homo-hexamer | 0.23 | HHblits | X-ray | 3.30 | 0.36 | 0.81 | LECTIN SCAFET PRECURSOR |
| 1dlp.1.D | 33.18 | homo-hexamer | 0.22 | HHblits | X-ray | 3.30 | 0.36 | 0.81 | LECTIN SCAFET PRECURSOR |
| 1dlp.1.A | 34.74 | homo-hexamer | 0.27 | BLAST | X-ray | 3.30 | 0.37 | 0.81 | LECTIN SCAFET PRECURSOR |
