# Supplementary material for: Discovery of antitumor lectins from rainforest tree root transcriptomes
Source: PLoS One. 2020 Feb 25;15(2):e0229467. doi: 10.1371/journal.pone.0229467 (PMC7041804; doi:10.1371/journal.pone.0229467)
Supplement: S2 Table — (DOCX) [file pone.0229467.s011.docx]

S2 Table. Glycan array fluorescent signal intensity.^a^

| Glycan in Figure 5  (from the top) | RayBio^®^ Glycan ID | Position on the Array  (column/row) | Normalized Background Subtracted Median Intensity | | Relative Normalized Spot Intensity  (Lectin minus Control)^b^ | Student’s t-Test  P-value |
| --- | --- | --- | --- | --- | --- | --- |
|  |  |  | Lectin Experimental Array Block  (Lectin and Ab) | Control or Reference Array Block (Ab only) |  |  |
| A1 | N024 | 16/13 | 613 | 163 | 450 | 0.00014 |
|  |  | 17/13 | 617 | 141 | 477 |  |
|  |  | 18/13 | 586 | 124 | 462 |  |
| A2 | N021 | 7/13 | 875 | 182 | 693 | 0.00068 |
|  |  | 8/13 | 822 | 200 | 622 |  |
|  |  | 9/13 | 875 | 176 | 698 |  |
| A3 | N030 | 7/14 | 1372 | 206 | 1165 | 0.00016 |
|  |  | 8/14 | 1289 | 176 | 1112 |  |
|  |  | 9/14 | 1335 | 156 | 1179 |  |
| A4 | N020 | 4/13 | 1593 | 216 | 1377 | 0.00093 |
|  |  | 5/13 | 1451 | 264 | 1188 |  |
|  |  | 6/13 | 1560 | 240 | 1320 |  |
| A5 | TE002 | 25/15 | 305 | 168 | 137 | 0.00043 |
|  |  | 26/15 | 328 | 145 | 183 |  |
|  |  | 27/15 | 280 | 97 | 183 |  |
| Not Shown | TE006 | 10/16 | 172 | 85 | 86 | 0.00083 |
|  |  | 11/16 | 219 | 120 | 99 |  |
|  |  | 12/16 | 108 | 13 | 95 |  |
| Not Shown | N210 | 10/14 | 195 | 136 | 58 | 0.00273 |
|  |  | 11/14 | 211 | 144 | 67 |  |
|  |  | 12/14 | 190 | 138 | 52 |  |
| Not Shown | G0075 | 16/9 | 431 | 401 | 30 | 0.03990 |
|  |  | 17/9 | 429 | 341 | 89 |  |
|  |  | 18/9 | 428 | 375 | 53 |  |
| Not Shown | G0077 | 22/9 | 488 | 457 | 26 | 0.07625 |
|  |  | 23/9 | 478 | 435 | 43 |  |
|  |  | 24/9 | 575 | 461 | 115 |  |
| B1 | N025 | 19/13 | 80 | 114 | -34 | 0.03166 |
|  |  | 20/13 | 57 | 122 | -65 |  |
|  |  | 21/13 | 85 | 115 | -20 |  |
| B2 | N022 | 10/13 | 81 | 110 | -29 | 0.05762 |
|  |  | 11/13 | 102 | 115 | -13 |  |
|  |  | 12/13 | 78 | 132 | -54 |  |
| B3 | N023 | 13/13 | 114 | 168 | -54 | 0.00453 |
|  |  | 14/13 | 125 | 191 | -66 |  |
|  |  | 15/13 | 89 | 164 | -76 |  |
| B4 | N040 | 13/14 | 77 | 169 | -92 | 0.01398 |
|  |  | 14/14 | 88 | 143 | -55 |  |
|  |  | 15/14 | 51 | 152 | -102 |  |
| B5 | G0100 | 10/12 | 535 | 838 | -303 | 0.00175 |
|  |  | 11/12 | 631 | 878 | -246 |  |
|  |  | 12/12 | 505 | 777 | -272 |  |

^a^Fluorescent signal intensity of spots within an array block was normalized according to the equation X(Ny) = X(y) * P1/P(y), where P1 = mean background corrected signal intensity of positive control spots on the reference array block, P(y) = mean background corrected signal intensity of positive control spots on array block “y”, X(y) = mean signal intensity for spot "X" on array block "y", X(Ny) = normalized signal intensity for spot "X" on array block "y". This normalized signal intensity value was used in calculating the probability associated with a Student's t-Test comparing the ability of a glycan on the experimental array block to hybridize TTRM_L1 with the same glycan on reference array block (to which only antibody was applied).

^b^Instances where the relative normalized spot intensity is negative suggests that the antibody recognized the glycan more strongly than that of the lectin.
