# Supplementary material for: Discovery of antitumor lectins from rainforest tree root transcriptomes
Source: PLoS One. 2020 Feb 25;15(2):e0229467. doi: 10.1371/journal.pone.0229467 (PMC7041804; doi:10.1371/journal.pone.0229467)
Supplement: Attachment — Submitted filename: Response to Reviewers.docx [file pone.0229467.s012.docx]

January 15, 2020

Dear Dr. Chammas:

Please find enclosed our revised manuscript, which during revision was re-titled to: “**Discovery of antitumor lectins from rainforest tree root transcriptomes**”. This manuscript was originally submitted under manuscript number: PONE-D-19-29568.

The manuscript received two reviews that were generally positive, with additional revisions requested to further support the conclusions of the paper. We have completed the requested changes, which we feel significantly strengthen the manuscript and now address the reviewer’s concerns. We list below the reviewers’ comments and our responses. Changes in the revised manuscript have been highlighted in yellow. We hope that the referees find our responses satisfactory and now consider the manuscript suitable for publication in PLoS One.

**Amended Funding Statement:**

The ML6 sequence was obtained and characterized using support from NSF DEB-1120476 and DEB-1457571, from material covered under export permits and a materials transfer agreement from Panama. This work was generously supported by a Charles E. Kaufman Foundation - New Initiative Award (KA2017-91785). There was no additional external funding received for this study.

| **Reviewer 1**  This manuscript describes studies on a novel lectin named TTRM_L1 that displays cellular toxicity toward a number of cell lines. The paper is preliminary in many ways, and should be significantly revised. |  | **Response-Reviewer 1**  We thank the reviewer for their careful critique of our work. Based on the referee’s comments we have performed a number of additional experiments. We feel the results of these experiments now provide more rigorous support of our conclusions and further strengthen the paper, as discussed below. |
| --- | --- | --- |
| **1.** The specificity of cellular cytotoxicity should be expanded to include non-tumor cell lines, such as primary fibroblasts and human lymphocytes stimulated to divide. |  | 1. As requested, we have now tested the toxicity of the lectin, which during revision has been renamed to ML6, against three primary non-cancerous cell lines: HDF (human dermal fibroblasts), NL20 (human lung epithelium) and HUVEC (human umbilical vein endothelial cells). We selected these lines based on the potent activity of ML6 against lung cancer cells. Hence, investigating off-target effects of the lectin against primary lung epithelium and vascular endothelial cells (assuming pre-clinical dosing would occur via the intravenous route) was prioritized.   Results in Fig. 3A and Table 1 of the revised manuscript, which are summarized below for convenience, now show that ML6 is also weakly active towards NL20 and HUVEC cells at the tested concentrations. |
|  | | |
| **2.** The specificity of the lectin and its binding to glycans appears to be broad and certainly not to structures that are unique to tumor cells. |  | 2. Our microarray results (Fig. 5A) show that ML6 selectively binds to only 5 glycans among an array of 300 different human structures, suggesting high target specificity. The referee’s comments may be related to the fact that the lectin’s putative target hapten, GlcNAc-β-1,2-Man-α-1,3-Man-β-1,4-GlcNAc-β-1,4-GlcNAc, is conserved across all human glycans. To better clarify this point, we have revised our discussion to describe that the tumor-specificity of ML6 may not be due to a unique target hapten, but more likely that this motif is more accessible on aberrant and incomplete glycans found on the surfaces of malignant cells. Conversely, this conserved motif is sterically shielded on ‘healthy’ hyperbranched glycan structures capped with terminating sialic acid residues. Supporting this assertion is our finding that the lectin did not bind to glycans with identical carbohydrate backbones, but which were terminated with sialic acid (see Fig. 5B). For convenience the revised figure is shown below. |
|  | | |
| **3.** The authors should assess cell binding independently of cell killing using the conventional flow cytometric or fluorescent assays commonly used. Does the lectin bind to all cells tested? |  | **3.** We appreciate the reviewer’s suggestion to more thoroughly assess cell binding. In our original submission, we utilized fluorescent confocal microscopy to evaluate ML6 binding to the lectin-sensitive lung cancer cell line A549 (Fig. 4). We now include results from similar studies investigating localization of ML6 in the insensitive cancerous (MCF7) and non-cancerous (NL20) cell lines. Micrographs shown in revised Fig. 4, which is reproduced below for convenience, demonstrates that ML6 preferentially binds to the lectin-sensitive cell line (A549) compared to insensitive controls (MCF7, NL20). Quantitation of cellular fluorescence from these images demonstrates that ML6 has >3 times the avidity for A549 cells than MCF-7 (Figure 4D). This cell-dependent surface binding may explain why ML6 shows selective killing of certain cell lines tested in our cytotoxicity panel (Figure 3A). |
|  | | |
| **4.** Is binding and killing by the lectin inhibited by carbohydrate-based haptens? This is important, as it would provide more assurance that carbohydrate binding to cells is essential to cell killing observed. |  | **4.** As requested, we have performed competitive inhibition assays using the monosaccharide hapten mannose. Results from these assays are reproduced below and included in Supplementary Figure 5 of the revised manuscript. This data demonstrates that lectin toxicity is significantly impaired by the presence of mannose in solution, with the addition of 1 mM mannose to the culture media resulting in a more significant decline in ML6 potency compared to 0.1 mM. This suggests that mannose haptens can inhibit ML6 anticancer activity in a dose-dependent manner, presumably via competitive binding with the lectin in solution. |
|  | | |
| **5.** The word hapten is not used, neither is carbohydrate inhibition, which is really unusual for a paper describing a lectin that binds to cells and kills them. |  | **5.** We thank the reviewer for bringing this oversight to our attention, and apologize for the error. Our understanding is the term hapten applies to small carbohydrates, generally mono- and disaccharides. We have now included this term in our discussion of the carbohydrate inhibition studies described above, and shown in Supplementary Figure 5. |
| **6.** The mechanism of cell killing by the lectin is not clear, and the authors should provide specific evidence in that regard. Do the cells undergo apoptosis? The authors do not explicity state this one way or the other? |  | **6.** We agree with the referee that the mechanism of ML6-mediated toxicity is important to investigate. Therefore, we have performed PI-Annexin V flow cytometry apoptosis assays – the results of which are now detailed in revised Figure 3, panels D&E and shown below for convenience. These studies demonstrate that, shortly after addition of the lectin (1 hour), cells display an apoptotic phenotype characterized by significant Annexin-V and PI co-staining. At 14 hours of incubation a considerable fraction of the treated cells occupy the quadrant indicative of late apoptosis. Taken in combination with our cell binding studies (Figure 4), this suggests that ML6 rapidly elicits its anticancer activity by binding to cell surface glycans and inducing apoptotic signaling cascades. |
|  | | |
| **7.** Does the lectin arrest cell division? Does the lectin cause cellular lysis? The authors also imply that the lectin inhibits the growth of a plant fungus, which is hardly a tumor cell. So the specificity of the lectin for killing tumor cells is a bit of a stretch for the title and the conclusions. |  | **7.** As described above, revision experiments demonstrate that ML6 mediates cell killing via an apoptotic mechanism (Figure 3D & E). During these studies we did not observe significant cell necrosis, suggesting the lectin is mediating apoptotic signaling to kill cancer cells rather than via membrane lysis.  The referee is correct that the lectin displayed both anti-fungal and anti-cancer activity. We decided to rigorously characterize the latter in this paper. Based on the antitumor properties of the lectin we feel the title of the manuscript is appropriate. However, to address the reviewer’s concerns we have now softened the language throughout the revised manuscript with respect to the specificity of the lectin for cancer cells. |
| **8.** What are the mono/disaccharide haptens of the lectin? This is another common parameter typically assessed for novel lectins. |  | **8.** We now include ITC results characterizing the binding affinity of ML6 with monosaccharide mannose haptens. This data is shown in the revised Supplementary Figure 2 and included below for the reviewer’s convenience.  Because lectin binding to monosaccharides is generally weak (Lee et al. *Glyconjugate J.*, 2000, 17), we prioritized additional experiments to analyze lectin binding towards complex mammalian glycans. This was done through microarray experiments, the results of which are shown in Figure 5. |
|  | | |
| **9.** The name of the lectin used in this paper is awkward, in that it has an underline in it. Moreover, the name should be the name of the genus/species or some idea of the organism making it? The concept that it is merely cloned and expressed by an unknown organism further illustrates the preliminary nature of the paper. |  | **9.** As suggested, the lectin has now been renamed to ML6 (Mannose-binding Lectin 6). Regarding the identity of the parent organism, the lectin was identified from bioinformatic analysis of complex root samples that contain a diverse array of microorganisms. The original specimens were obtained from tropical tree roots isolated in Panama. Our limited access to the original material, taken together with the complex ecologic nature of the specimens, means the identification of the exact producing organism is impractical.  That said, the referee’s comments have made us aware that we did not sufficiently describe in the manuscript how the lectin sequence was bioinformatically identified and selected. We now include a detailed description of this in the revised materials and methods section. |
|  |  |  |
| **Reviewer 2**  The manuscript entitled "Discovery of antitumor lectins from competing microorganismal ecologic niches" by Lawanprasert et al. describes the discovery of a engineered lectin with selectivity for cancer carbohydrate antigens and cause cell death. Overall this is an excellent manuscript worthy of publication in PLOS ONE. The description of a novel very-potent anticancer protein is of significant interest. The paper could be improved with minor revision. |  | **Response-Reviewer 2**  We thank the reviewer for their positive feedback of our work. Based on the referee’s comments we have included additional data and supporting references in the manuscript that we feel now further strengthen the conclusions of the paper, as discussed below. Please be advised that based on reviewer comments we have changed the name of our lectin from TTRM_L1 to ML6. |
| **1.** The introduction could be improved with better referencing of statements ( i.e "lectins are found in organismal proteomes, lipides and secretes" - referees for each category should be included). |  | **1.** We now include additional references in the revised introduction to better support our statements, including the passage highlighted by the referee. |
| **2.** Some of the language in the introduction is too casual. For example the opening sentence of paragraph 3 states " Nature contains a much broader diversity of lectins than present in any single species genome" - this is obvious and is followed by a statement on therapeutic potential that does not rely on the opening statement. |  | **2.** As requested we have modified the introduction to remove superfluous passages and improve the clarity of our background discussion. |
| **3.** The abbreviation MBL should not be used in a general sense as it is generally used only to refer to mannose binding lectin in human plasma. It should not be used as an acronym for all mannose-specific lectins such as, I believe, griffithsin in one sentence followed by a reference to "the" MBL in the next sentence two references for different proteins are both being referred to by the same abbreviation. |  | **3.** We apologize for the confusion and have corrected the text to only use the abbreviation MBL when discussing the human mannose binding lectin. |
| **4.** The last sentence of paragraph 3 also seem a bit of a throw away line. No other reference to toxin conjugates and no real attachment to the rest of the paragraph. |  | **4.** We have removed this sentence in the revised manuscript. |
| **5.** Results:  The method by which the authors identified lectins in the genome should be discussed as this is not common knowledge. |  | **5.** As requested we have updated the materials and methods with an additional section to more clearly describe how the lectin gene sequence was identified and selected. |
| **6.** The authors state that due to the varied species and interdependence of rainforest communities suggests that many of the 80 lectins identified "are" involved in between-species interactions. - There is no reason to expect so unless they can also show these proteins are secreted or are surface expressed. Stating that some of these proteins could be involved in such interactions seems more accurate. |  | **6.** We agree with the referee and have now softened the language used in the revised passage. In addition, we have changed the title of the manuscript to avoid confusion on this point. |
| **7.** The models produced for figure 2 are just that, speculative models. Paper could be significantly improved with actual structures. |  | **7.** The reviewer’s suggestion is well taken, and we agree that defining a crystal structure for the lectin would be helpful. In fact, after submission of our original manuscript draft, we immediately begun studies with the X-ray crystallography facility at Penn State to develop crystals of the recombinant protein. Unfortunately, despite numerous iterations and testing an array of experimental conditions, we have not been able to obtain stable crystals of sufficient size before the submission deadline of our revised manuscript (Jan 5^th^). We will continue to optimize conditions towards obtaining stable ML6 crystals and publish the results of these experiments in due course. |
| **8.** Results could be improved with studies on agglutination and /or cell permeability changes associated with treating cells with TRRM1. Also confocal studies would be improved by including insensitive cells to see differences in TRRM location with time. |  | **8.** We now include additional results on cell agglutination by ML6. These studies show that incubation of the lectin-sensitive cell line, A549, with ML6 does not potentiate any significant changes in cell clustering. Results of these studies are shown in Supplementary Figure 8 of the revised manuscript, and are reproduced below (top). In these experiments we measure average cluster area of cell aggregates from microscopy images of A549 cells before (black bar) and after (open bar) incubation with 10 μM ML6.  In addition, we now include results from fluorescent confocal studies investigating localization of ML6 in the insensitive cancerous (MCF7) and non-cancerous (NL20) cell lines. Micrographs shown in revised Fig. 4, which is also reproduced below for convenience (bottom), demonstrates that ML6 preferentially binds to the lectin-sensitive cell line (A549) compared to insensitive controls (MCF7, NL20). Quantitation of cellular fluorescence from these images demonstrates that ML6 has >3 times the avidity for A549 cells than MCF-7 (Figure 4D). This cell-dependent surface binding may explain why ML6 shows selective killing of certain cell lines tested in our cytotoxicity panel (Figure 3A). |
|    | | |
| **9.** Figure 5 would be improved by including a legend fo monosaccharide identities for readers not familiar with the nomenclature of oligosaccharide structure. |  | **9.** We have updated Figure 5 to include the requested legend. |
| **10.** paper could be improved by inclusion of direct binding studies ( with quantitation) between select oligosaccharides and TRRM1. ITC, SPR etc would be good. |  | **10.** We now include ITC results in the revised supplementary information characterizing the binding affinity of ML6 with monosaccharide mannose. This data is shown in the revised Supplementary Figure 2 and included below for the reviewer’s convenience.  We did consider additional ITC studies to quantitate the binding affinity of ML6 with the putative glycan targets identified during the microarray assays. However, custom synthesis of these oligosaccharides at the scale needed for replicate binding assays was unfortunately cost prohibitive. |
|  | | |
| **11.** Any evidence of aggregation in TRRM1? Is the expressed protein mono disperse? Some evidence of the purity and identity of the expressed protein would be useful to see at least in the supplemental data. LC/MS sequencing, SDS-Page... |  | **11.** The ML6 lectin was stable in buffer for up to one week if stored at 4°C. For our studies, aliquots of ML6 were used within three days of being thawed. This language has been added to the materials and methods section. We did observe the formation of protein flocculates if the lectin was stored for longer than one week at 4°C; which is why we made sure to use the protein quickly after thawing.  In regards to protein purity, we evaluated this using SDS-page, the results of which are now shown in the revised Supplementary Figure 1 and included below for convenience. |
|  | | |
| **12.** Discussion:  The discussion should be better referenced. Several statements in the opening paragraph should be referenced. |  | **12.** We thank the reviewer for bringing this oversight to our attention. We have now appropriately referenced our discussion section in the revised manuscript. |
| **13.** The speculation in paragraph 2 on mechanism, based solely on the model of protein structure are weak. Better mechanism discussion would be achieved by additional experimental data on extrinsic vs. intrinsic pathways and data on apoptosis ve. necrosis, membrane binding differences between sensitive and resistant cells, etc. |  | **13.** In Figure 3 of the revised manuscript (new data shown below) we now include results from apoptosis assays performed on A549 cells with and without treatment of ML6. These studies demonstrate that, shortly after addition of the lectin (1 hour), cells display an apoptotic phenotype characterized by significant Annexin-V and PI co-staining. At 14 hours of incubation a considerable fraction of the treated cells occupy the quadrant indicative of late apoptosis. Taken in combination with our cell binding studies (Figure 4), which shows that ML6 preferentially binds to lectin-sensitive cells, this suggests that the lectin rapidly elicits its anticancer activity by binding to cell surface glycans and inducing apoptotic signaling cascades.  Based on this new data, we have modified the passage in question to: “…This assertion is collectively supported by our data showing that ML6 consists of a β-sheet rich structure (**Figure 2**) like that of many antitumor legume lectins, elicits its cytotoxic effects at incredibly low concentrations (**Table 1**) and induces apoptotic cellular phenotypes over a time period commensurate with its membrane and cytoplasmic localization (**Figures 3 & 4**).” |
|  | | |
| **14.** Additional speculation on a ER-driven mechanism would be enhanced by better timelines of cytotoxicity and looking directly at ER flux in TRRM1 treated cells. Such experiments would improve this manuscript and the discussion. |  | **14.** We agree with the reviewer that experiments to probe the potential for ER-driven cytotoxic mechanisms of ML6 are interesting. However, in light of new results from our apoptosis assays - which now provide a temporal link between cellular localization of the lectin and induction of apoptosis – we have modified our discussion to avoid speculating on ER-dependent mechanisms. |
| **15.** Overall the manuscript is worthy of publication but could be improved by a few select experiments to better define mechanism and a a bit more data ( purity analysis of recombinant TRRM1, confocal controls with resistant cells). |  | **15.** We thank the reviewer for their important insights. Based on the comments provided we have performed a number of additional experiments, as described above, to investigate the cellular localization of the lectin and mechanisms of cell-specific toxicity, among others. We feel this new data supports the assertions in the paper and further strengthens the conclusions of our work. We hope the referee now finds the manuscript suitable for publication. |
